# Review on Chamber-Specific Differences in Right and Left Heart Reactive Oxygen Species Handling

**DOI:** 10.3389/fphys.2018.01799

**Published:** 2018-12-17

**Authors:** Klaus-Dieter Schlüter, Hanna Sarah Kutsche, Christine Hirschhäuser, Rolf Schreckenberg, Rainer Schulz

**Affiliations:** Department of Physiology, Justus-Liebig-University Giessen, Giessen, Germany

**Keywords:** cardiac remodeling, heart failure, oxidative stress, pulmonary hypertension, uncoupling protein, MAO

## Abstract

Reactive oxygen species (ROS) exert signaling character (redox signaling), or damaging character (oxidative stress) on cardiac tissue depending on their concentration and/or reactivity. The steady state of ROS concentration is determined by the interplay between its production (mitochondrial, cytosolic, and sarcolemmal enzymes) and ROS defense enzymes (mitochondria, cytosol). Recent studies suggest that ROS regulation is different in the left and right ventricle of the heart, specifically by a different activity of superoxide dismutase (SOD). Mitochondrial ROS defense seems to be lower in right ventricular tissue compared to left ventricular tissue. In this review we summarize the current evidence for heart chamber specific differences in ROS regulation that may play a major role in an observed inability of the right ventricle to compensate for cardiac stress such as pulmonary hypertension. Based on the current knowledge regimes to increase ROS defense in right ventricular tissue should be in the focus for the development of future therapies concerning right heart failure.

## Introduction

**Figure 1:**
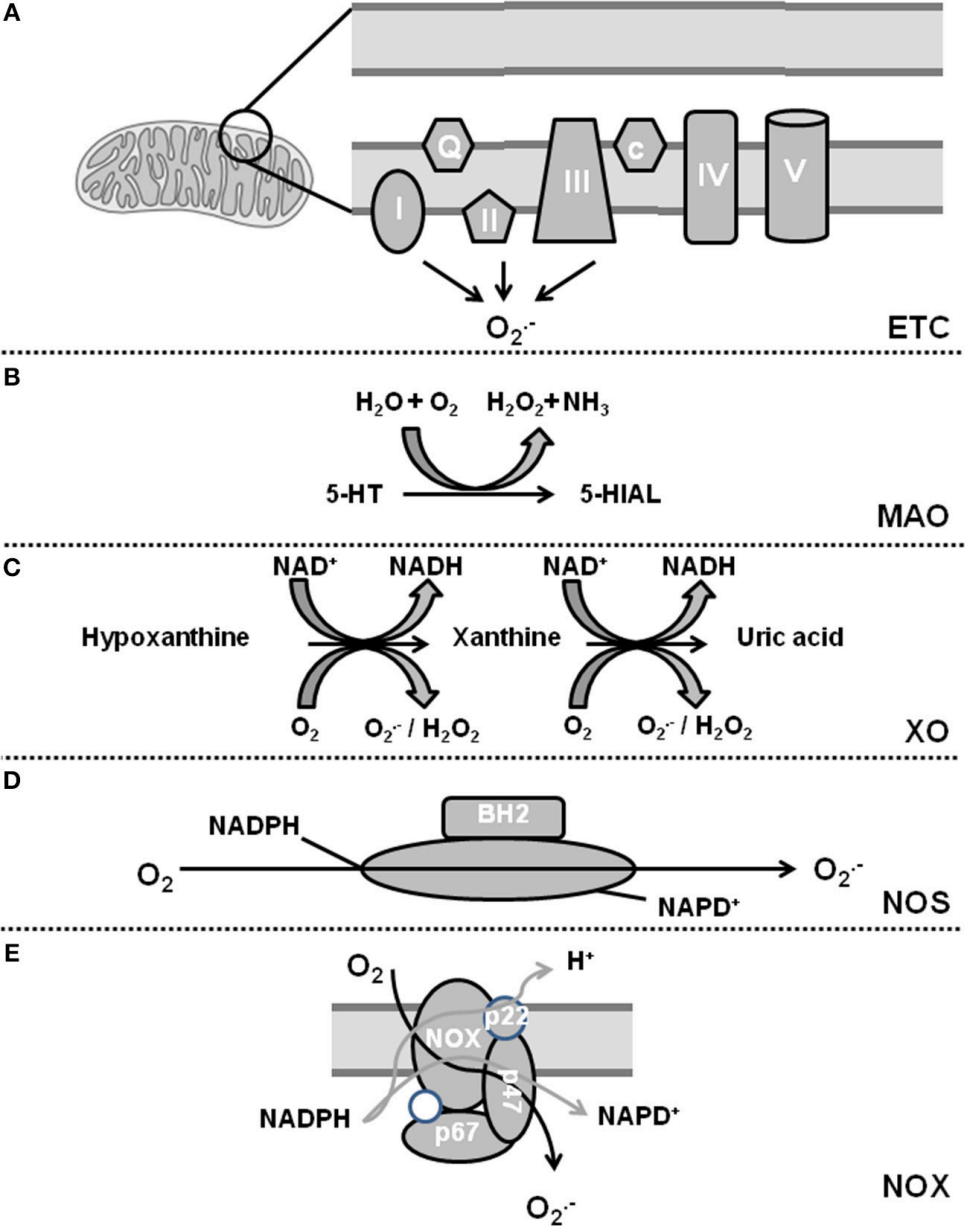
Sources of reactive oxygen species in cardiomyocytes. **(A)** Complex I and III of the electron transport chain (ETC) constitutively release ${\text{O}}_{2}^{.-}$ and complex II can be activated by NOX-dependent ROS. **(B)** Monoamine oxidase (MAO) generates H_2_O_2_. **(C)** Xanthine oxidase (XO) catalyzes a two-step reaction leading to additional release of H_2_O_2_. **(D)** Uncoupling of nitric oxide synthase (NOS) leads to generation of ${\text{O}}_{2}^{.-}$. **(E)** NADPH oxidase (NOX) generates also ${\text{O}}_{2}^{.-}$ upon activation.

**Figure 2:**
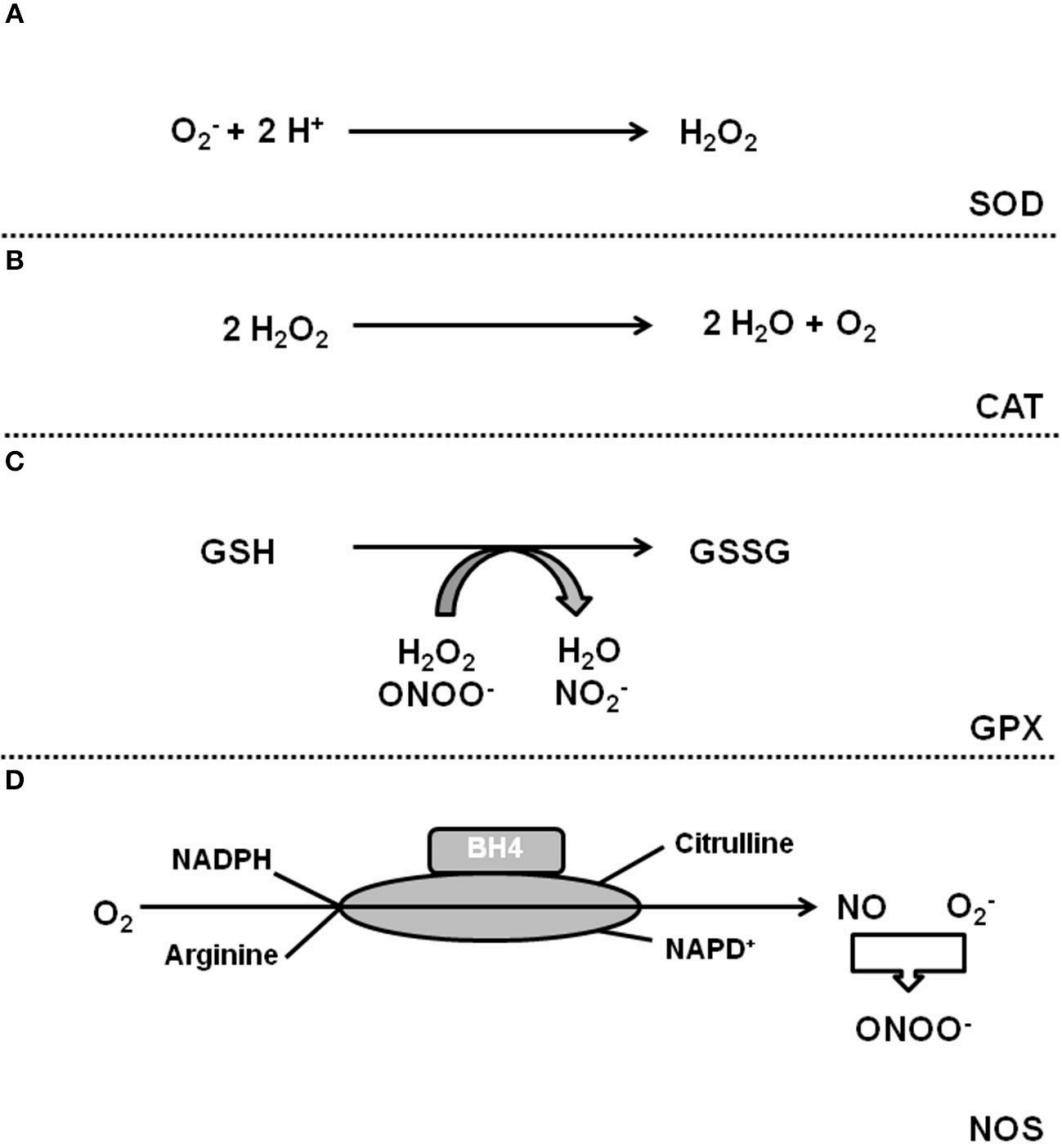
ROS defense systems in cardiomyocytes. **(A)** Superoxide dismutases catalyze the formation of H_2_O_2_ that can be detoxificated by catalase **(B)**. **(C)** Gluatathione peroxidase (GPX) reduces H_2_O_2_. **(D)** Coupled NOS generates NO that neutralizes ${\text{O}}_{2}^{.-}$.

Oxidative stress is defined as a condition by which an imbalance occurs between the production of reactive oxygen species (ROS) and the antioxidant defense system. The term ROS includes molecules that have one or more unpaired electrons (i.e., superoxide and hydroxyl) and non-radicals that are able to generate free radicals (i.e., hydrogen peroxide). Intracellular sources of ROS are the electron transport chain of the mitochondria, monoamine oxidase (MAO), p66shc (for review, see Di Lisa et al., 2017), xanthine oxidase (XO), uncoupling proteins (UCP, depending on the mitochondrial membrane potential; for review see Cadenas, 2018), uncoupled nitric oxide (NO) synthase (NOS), sodium-potassium ATPase (NKA), and nicotinamide adenine dinucleotide phosphate (NADPH) oxidase (NOX) (for review, see Egea et al., 2017). The defense system contains enzymes such as superoxide dismutase (SOD), catalase, glutathione peroxidase, and coupled NOS. Figures 1, 2 give an overview about ROS sources and ROS defense systems in the heart. Whereas subtle changes in ROS act as intracellular signaling pathways (*redox signaling*) high levels of ROS can cause cell damage and dysfunction (*oxidative stress*) (for review, see Egea et al., 2017).

The following review article now summarizes our current understanding about similarities and differences in ROS handling between LV and RV. We searched the current literature (PubMed, MedLine data bank until July, 2018) using the terms “right heart and ROS,” “pulmonary hypertension and ROS,” “RV failure and ROS,” “LV failure and ROS,” “RV hypertrophy and ROS” and “LV hypertrophy and ROS.” Most studies dealing with ROS and RV hypertrophy used models of pulmonary hypertension induced by banding, monocrotaline injection or chronic hypoxia. In these models ROS contribute to pulmonary hypertension and RV remodeling. In many studies, effects of ROS reduction on RV hypertrophy were secondary to reduced pulmonary pressure (reviewed by Wong et al., 2013). In the current review we therefore focus on studies directly assessing ROS and ROS-dependent effects in RV tissue and compare these results to established concepts generated from the LV.

## Oxidative Stress in the Heart

Oxidative stress in cardiomyocytes occurs during chronic pressure or volume overload of the heart (Gladden et al., 2013; Hansen et al., 2016), cardiac ischemia/ reperfusion (Riba et al., 2017), cardiomyopathy (Ishikawa et al., 2005), diabetes (Guido et al., 2017), chemotherapy-induced heart failure in the left (Mouli et al., 2015; Li et al., 2018), and right ventricle (Anghel et al., 2018), poison such as cigarette smoke (Talukder et al., 2011), chronic kidney disease (Duni et al., 2017), or aging (Chang et al., 2017), or as a response to congenital heart failure (Iacobazzi et al., 2016). Within the heart other sources of ROS are cells adjacent to cardiomyocytes such as inflammatory cells (Xu et al., 2011; Hernandez-Resendiz et al., 2018), endothelial cells (Burger et al., 2011), stem cells (Mandraffino et al., 2017), and cardiac fibroblasts (Ciulla et al., 2011). Redox signaling contributes to cardiac hypertrophy and even more important oxidative stress contributes to the transition of adaptive to maladaptive cardiac hypertrophy, named maladaptive remodeling. Oxidative stress can damage cells by growth factor-independent activation of cardiac growth regulation (Calamaras et al., 2015), can inactivate NO leading to loss of myocyte-specific NO function (Rassaf et al., 2006; Lüneburg et al., 2016), can directly reduce cardiomyocyte function by oxidative modification of sarcomere proteins such as tropomyosin (Heusch et al., 2010b; Canton et al., 2011) or sarcoplasmatic reticulum proteins (i.e., SERCA; Qin et al., 2017), can induce a calcium desensitization of myofibrils (Wang et al., 2008), can activate the Na-K-ATPase (Wang et al., 2017a), can damage mitochondrial function (Ide et al., 2001; Sverdlov et al., 2016), or can induce cell death (apoptosis, necrosis; Redza-Dutordoir and Averill-Bates, 2016). Therefore, ROS defense strategies of the cells are necessary for cell survival and functional stabilization in both ventricles.

## Differences Between Right and Left Ventricle

**Figure 3:**
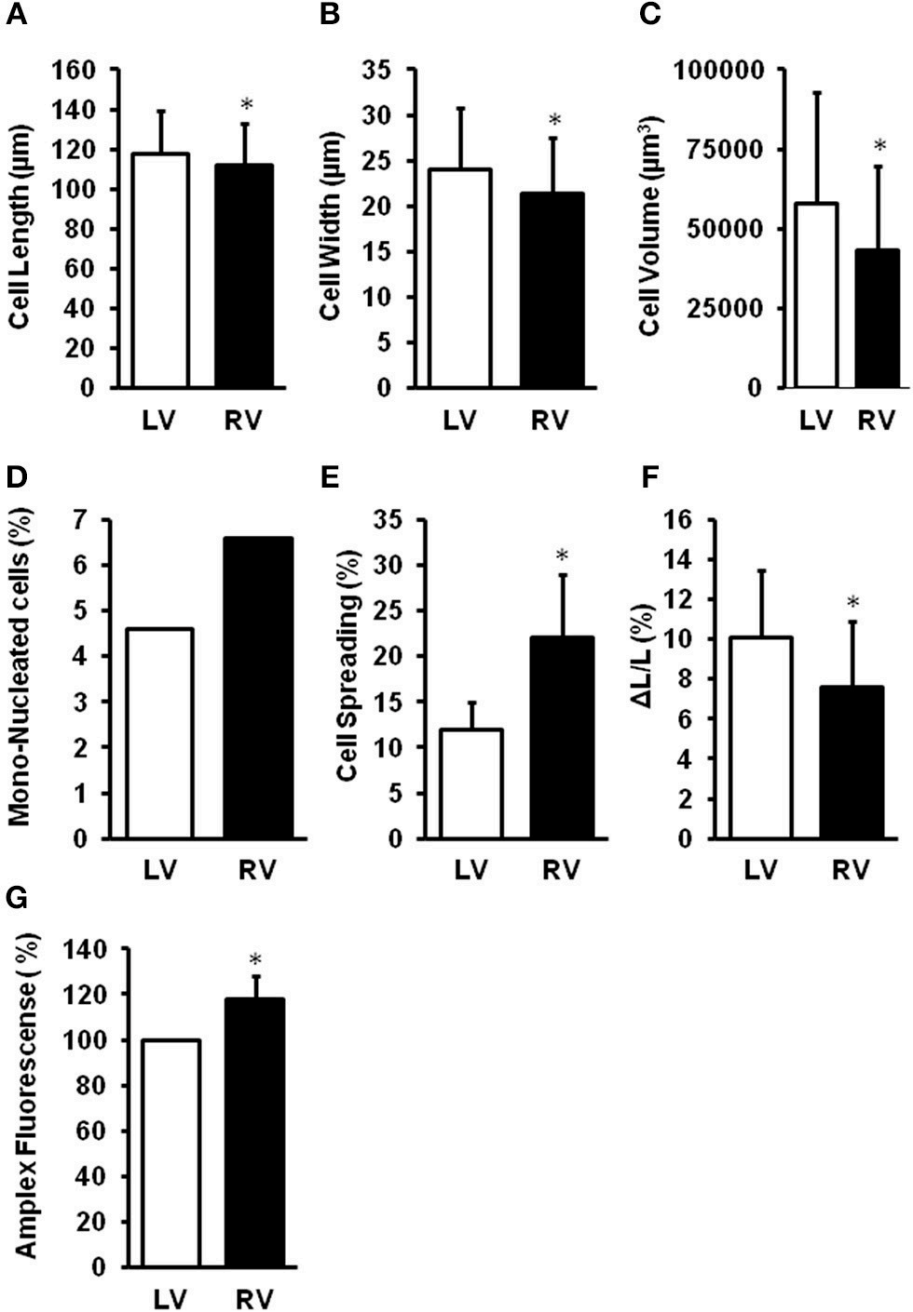
Differences between right (RV) and left (LV) ventricular cardiomyocytes. LV cardiomyocytes are longer **(A)**, wider **(B)**, larger **(C)**, less mono-nucleated **(D)**, have a reduced cell spreading **(E)** as adaptation to culture conditions, and a stronger load-free cell shortening **(F)**. Furthermore, mitochondria from RV generate more ROS **(G)**. Data depicted from Schreckenberg et al. (2015) and Schlüter (2016). *p < 0.05 vs. LV.

The different chambers of the heart are derived from different embryonic origin, namely the first (left ventricle, LV) and second heart field (right ventricle, RV). In rodent hearts, cardiomyocytes isolated from the LV or RV differ in size, number of mono-nucleated cells, cellular adaptation to culture conditions, and cell shortening (Schlüter, 2016; summarized in Figure 3). This gives raise to speculations that the LV and RV may differentially respond to cardiac stresses. Pressure overload is associated with adaptations performed on the transcriptional level. Many of them are similar between the RV and LV. However, some genes are up-regulated in the pressure-overloaded RV only, including genes involved in Wnt signaling (Dickkopf 3, Sfrp2, and Wif1), annexin A7, clusterin/apolipoprotein J, neuroblastoma suppression of tumorigenicity 1 (Nbl1), formin-binding protein (Fnbp4), and the lectin-like oxidized low-density lipoprotein (oxLDL) receptor (LOX; Reddy and Bernstein, 2015). Differences occur also on the level of miRNA (Reddy and Bernstein, 2015). Considering the high impact of ROS for cardiac adaptation to pressure overload, it is also important to understand such differences with respect to ROS formation, ROS defense, and ROS-dependent cellular responses. Indeed, mitochondria isolated from the LV or RV of rat hearts generates different amounts of ROS (Schreckenberg et al., 2015; summarized in Figure 3). Treatment of isolated perfused rat hearts with serotonin, a substrate for MAO, results in the promotion of protein carbonylation as evidenced by increased ROS formation, specifically in the RV but not the LV. Interestingly, no differences between RV and LV antioxidant enzymes and serotonin receptors/transporter are detected (Liu et al., 2008).

## ROS in Right Heart Hypertrophy and Failure: Cytosolic ROS

Pressure overload induces the NOX subunit gp91 (Li et al., 2002; Byrne et al., 2003; Tanaka et al., 2005; Grieve et al., 2006; Liu et al., 2006, 2010; Guggilam et al., 2007; DeMarco et al., 2008; Nisbet et al., 2009; Chemaly et al., 2011; Xu et al., 2011; Ogura et al., 2013; Frazziano et al., 2014; Matsuda et al., 2015; Ma et al., 2016; Sirker et al., 2016; Zhu et al., 2017). In human right and left heart failure the p47phox subunit of NOX also translocates to the sarcrolemma (Nediani et al., 2007). Increased NOX expression is associated with increased formation of superoxide anions (Nediani et al., 2007; Ogura et al., 2013; Dos Santos Lacerda et al., 2017; Türck et al., 2018). Furthermore, hypoxia, leading to pulmonary hypertension, and RV hypertrophy, increases RV expression of NOX2/4 (Liu et al., 2014; Ye et al., 2016; Zhu et al., 2017). In the monocrotaline-induced pulmonary hypertension rat model, Nox4 expression is induced in cardiomyocytes but also in the intercellular area (mainly co-localizing with fibroblasts) (He et al., 2017). In the RV, NOX4 is also regulated by the α_1A_-receptor (Cowley et al., 2017); stimulation of this receptor decreases NOX4 expression during pulmonary hypertension. NOX-dependent ROS modifies mitochondrial function by increased release of ROS from complex II of the mitochondria during the transition from RV hypertrophy to failure (Redout et al., 2007) (ROS induced ROS release). There are differences between the RV and LV ventricles in the primary source of ROS production. In the RV, NOX, and mitochondrial complex II activity both increase during the transition to heart failure, whereas, in the LV, NOX appears to be the primary source of ROS generation (Redout et al., 2010).

Xanthine oxidoreductase (XO) activity remains unaltered in the monocrotaline-induced RV hypertrophy model but its activity increases with the transition to RV failure. XO is mainly localized in CD68^+^ inflammatory cells based on studies with an affinity-purified polyclonal antibody (de Jong et al., 2000). In another rat model of pulmonary hypertension-induced RV failure, metabolomics analysis revealed an increase in xanthine, and uric acid in the hypertrophied RV, suggesting the production of ROS by XO. Furthermore, the RV level of α-tocopherol nicotinate declined, consistent with oxidative stress decreasing antioxidants (Wang et al., 2017). XO also contributes to ROS formation in LV (Moris et al., 2017).

Uncoupled NOS is another protein involved in the generation of oxidative stress in the LV secondary to pressure overload (Takimoto et al., 2005). In the RV, uncoupled NOS contributes to ROS generation, too. In caveolin-1 (Cav-1) knockout mice subjected to chronic hypoxia the transition from RV hypertrophy to failure is accelerated compared to wild-type mice and caused by uncoupling of RV endothelial NOS and increased protein tyrosine nitration; all changes are prevented by re-expressing an endothelial-specific Cav-1 transgene (avoiding NOS uncoupling) or by NOS inhibition without modifying the extent of pulmonary hypertension (Cruz et al., 2012). Also, in a pharmacological model of hypertension chronic administration of L-NAME leads to uncoupling of NOS in RV (Schreckenberg et al., 2015).

Uncoupling of NOS can be caused by reduced substrate (arginine) for NOS. Reasons for substrate limitation can be an induction of arginase that leads to substrate limitation (Heusch et al., 2010; Schreckenberg et al., 2015), increased plasma concentrations of asymmetric dimethyl arginine (ADMA), a natural circulating inhibitor of NOS (Lüneburg et al., 2016), or depletion of NOS with tetrahydrobiopterin (BH_4_, Shimizu et al., 2013).

## ROS in Right Heart Hypertrophy and Failure: Mitochondrial ROS

A proteomic analysis of the normal rabbit and porcine RV and LV free walls shows equivalent cellular aerobic capacity, volume of mitochondria, mitochondrial enzyme content (cytochrome c oxidase, respiratory complexes 1 and 3–5, aconitase, SOD), and mitochondrial enzyme activities (Phillips et al., 2011). Interestingly, mitochondrial membrane potential, a surrogate of overall mitochondrial function, is lower in the resting RV compared to the LV (Nagendran et al., 2008), while—at least in rats—ROS formation in mitochondria isolated from the RV is slightly higher than in LV mitochondria (Schreckenberg et al., 2015). At last in part, the latter might be the consequence of a reduced ROS defense capacity (Borchi et al., 2010; Manni et al., 2016). Comparing mitochondria from hypertrophic RV with those of non-hypertrophic LV revealed differences in electrone transport chain activity and ATP generating enzyme expression Gupto et al., 2016.

While the mitochondrial protein profiles of the RV and LV are quite similar at rest, they diverge when subjected to an increased afterload (Phillips et al., 2011), and mitochondrial membrane potential increases with RV hypertrophy (Nagendran et al., 2008). This hyperpolarization of mitochondria, indicating reduced oxidative phosphorylation, is related to an activation of the nuclear factor of activated T cells (NFAT) pathway and is reversed by dichloroacetate, an inhibitor of pyruvate dehydrogenase kinases (PDK) (Nagendran et al., 2008). Thus, an increase in PDK activity in RV hypertrophy contributes to the decreased oxidation of pyruvate in mitochondria and an increased conversion of pyruvate to lactate in the cytosol. An increase in glycolytic hexokinase and lactate dehydrogenase activities occurs following monocrotaline-induced pulmonary hypertension at the stage of compensated RV hypertrophy (Balestra et al., 2015), further supporting the concept of a metabolic switch from mitochondrial oxidative phosphorylation to glycolysis in the compensated phase of RV hypertrophy (Paulin and Michelakis, 2014; Sutendra and Michelakis, 2014). Indeed, a decreased mitochondrial oxygen usage and an increased anaerobic glycolysis has been described in patients with pulmonary hypertension (Wong et al., 2011) (for a detailed review, see Freund-Michel et al., 2014), and the decrease in mitochondrial oxidative phosphorylation during the development of RV hypertrophy has been suggested to decrease mitochondrial ROS formation (for review, see Paulin and Michelakis, 2014).

The increase in glucose uptake and the mitochondrial hyperpolarization are lost with the progression of RV hypertrophy to failure (Nagendran et al., 2008). For the LV, ROS sensors revealed increased mitochondrial ROS in resting and contracting cardiomyocytes during the progression to heart failure. Pathway analysis of mitochondrial ROS-sensitive networks indicated that increased mitochondrial ROS in failing cardiomyocytes disrupts the normal coupling between cytosolic signals and nuclear gene programs driving mitochondrial function, calcium handling, action potential repolarization, and antioxidant enzymes (Dey et al., 2018). Indeed, in the RV, during the transition from RV hypertrophy to RV failure, mitochondrial ROS defense system (SOD-2) is down-regulated (Redout et al., 2007).

Another key regulator that is decreased during RV failure is the peroxisome proliferator-activated receptor gamma coactivator (PGC) 1α, leading to impaired fatty acid oxidation, decreased mitochondrial mass and number, and reduced oxidative capacity, potentially contributing to increased ROS production (Karamanlidis et al., 2011; Gomez-Arroyo et al., 2013). In an animal model of pulmonary hypertension-induced RV failure, fatty acid oxidation decreases secondary to the failure of palmitoylcarnitine to stimulate oxygen consumption. In humans with pulmonary hypertension, RV long-chain fatty acids and triglyceride contents are increased and ceramide, a mediator of lipotoxicity, accumulates (Brittain et al., 2016).

## ROS Defense Systems in RV

In rats treated with monocrotaline to increase pulmonary artery pressure without inducing RV hypertrophy, RV hydrogen peroxide increases but SOD, catalase, and glutathione peroxidase activities are also enhanced (Siqueira et al., 2018).

During pressure overload–induced LV hypertrophy, antioxidant enzymes are activated in the compensated stage but their activity decreases during the onset of LV failure. In contrast, only the antioxidant enzyme catalase becomes activated in some (Ecarnot-Laubriet et al., 2003) but not all studies (Pichardo et al., 1999) while SOD and glutathione peroxidase are not activated at all in the compensated stage of RV hypertrophy secondary to pulmonary hypertension, predisposing the RV to ROS induced damage at an earlier stage than in the LV (Pichardo et al., 1999; Ecarnot-Laubriet et al., 2003; Schreckenberg et al., 2015). With a progression of from RV hypertrophy to failure, down-regulation of antioxidant enzymes, and increased ROS production occurs in a mice model of pulmonary hypertension (Aziz et al., 2015; Reddy and Bernstein, 2015), although in one model of monocrotaline-induced RV failure, glutathione peroxidase increases while catalase, and SOD activities are similar to sham animals (Türck et al., 2018).

Despite some controversial results the general view, however, remains that increased ROS formation and decreased ROS defense leads to increased oxidative stress driving the progression from RV hypertrophy to RV failure.

## Downstream Signaling

(Patho)physiological conditions known to activate p38 mitogen activated protein (MAP) kinase are often associated with increased ROS formation (Wenzel et al., 2001, 2006, 2007). Indeed, p38 MAP kinase is activated by oxidative stress (Redout et al., 2007). An activation of p38 MAP kinase pathways is linked to cardiac hypertrophy and dysfunction and in RV and LV of end-stage failing human hearts, p38 MAP kinase and extracellular-signal regulated kinase (ERK), but not c-Jun N-terminal kinases (JNK), are activated; a significant correlation between protein kinase activities is observed between LV and RV from the same heart (Nediani et al., 2007).

Increased ROS subsequently modifies tropomyosin, induces matrix metalloproteases (MMPs 2, 9, and 13), sensitizes β-adrenoceptors (via induction of protein kinase C-ε), and causes endothelial dysfunction in the right ventricle (Cheng et al., 2009; Lu et al., 2011; Cau et al., 2013; Schreckenberg et al., 2015).

In LV tissue, ROS is associated with an induction of p90^rsk^ and the sodium-proton-exchanger (NHE) and furthermore, via ROS-dependent formation of lipid peroxidation-derived aldehydes (Cingolani et al., 2011). Furthermore, ROS activates the mammalian target of rapamycin (mTOR)-p70^s6k^ pathway (Calamaras et al., 2015). Both pathways (NHE and mTOR-p70^s6k^) are also involved in growth factor-dependent acceleration of protein synthesis (Simm et al., 1998; Schäfer et al., 2002). Commonly ROS and growth factors activate also the ERK pathway but the latter is not directly linked to the regulation of protein synthesis (Pönicke et al., 2001; Calamaras et al., 2015).

Apart from NOX, activation of the renin-angiotensin-system is apparent in the RV during pressure overload (for review, see Ameri et al., 2016). Compared to normal hearts, however, angiotensin II binding is diminished in the failing RV of pulmonary artery hypertension patients due to angiotensin II type 1 receptor down-regulation, despite RV myocardial angiotensin converting enzyme (ACE) activity being increased (Zisman et al., 1998). Interestingly, the ACE DD genotype, associated with an increased myocardial ACE activity, is more frequent in patients with pulmonary hypertension than in healthy individuals, but it is also associated with preserved RV function in pulmonary hypertension patients (Abraham et al., 2003).

A specific role for LOX-1 in RV hypertrophy and failure has been suggested. First, oxLDL receptors cross react with NOX (Ogura et al., 2013). Furthermore, ventricular expression of oxLDL receptors is induced under hypoxia leading to pulmonary hypertension and RV hypertrophy (Zhu et al., 2017). Cross-reactivity of oxLDL receptors with angiotensin II receptors type 1 has also been reported. In all these cases, NOX is subsequently activated favoring oxidative stress. It seems that this mechanism plays an important role in right heart failure.

## Therapeutic Implications

In general, attenuation of mitochondrial-derived oxidative stress is a reasonable therapeutic concept to attenuate RV hypertrophy and transition to RV failure (for review, see Maarman et al., 2017).

As expected from the findings that ROS is increased in RV hypertrophy and transition to RV failure, trapping molecules targeting mitochondrial ROS (mitoTEMPO), folic acid, EUK-134, a synthetic antioxidant mimicking the activity of SOD, attenuate RV hypertrophy (Redout et al., 2010; Qipshidze et al., 2012; Datta et al., 2015).

Regulation of SOD, in particular SOD-1 (located in the cytosol), and SOD-2 (located in mitochondria), has been proven to attenuate hypertrophy and even more important transition to heart failure. In a pharmacological rat model of hypertension (L-NAME induced hypertension) SOD-2 was induced in the LV but not in the RV (Schreckenberg et al., 2015). Up-regulation of SOD-2 in the LV was associated with less oxidative stress and preserved function in the presence of hypertrophy. Similarly, induction of SOD-2 activity has repeatedly reported to improve cardiac function. Interestingly, at least for the LV multiple strategies to improve SOD activity work, such as the natural product Sheng-Mai-San (Chai et al., 2016), inhibition of the renin-angiotensin-system (Tanaka et al., 2005), calcium antagonists (Umemoto et al., 2004), or resveratrol (Danz et al., 2009). Whether any of these mechanisms is sufficient to increase SOD activity in RV remains unclear. As mentioned above, SOD is induced during hypertrophy in LV tissue (Date et al., 2002; Lu et al., 2010; Qiao et al., 2014; Aziz et al., 2015; Schreckenberg et al., 2015). Failure to increase SOD as an adaptive mechanism to rescue mitochondrial and cytosolic ROS is associated with heart failure (Redout et al., 2007; Koga et al., 2013). In a model of bronchopulmonary dysplasia, SOD-2 expression but not activity is induced leaving ROS formation unchanged. This underlines the importance of SOD-2 activity for protection against ROS-derived damage. Failure of the RV to up-regulate SOD-2 expression and activity might be a key step for right heart failure.

Other treatment using secoisolariciresinol diglucoside (Puukila et al., 2017), dehydroepiandrosterone (Alzoubi et al., 2013; Rawat et al., 2014), trimethoxystilbene (Liu et al., 2014), pterostilbene (Dos Santos Lacerda et al., 2017), trapidil (Türck et al., 2018), and α_1A_-adrenoceptor stimulation with A61603 (Cowley et al., 2017) and finally fenofibrate (Galhotra et al., 2018) attenuate both RV hypertrophy and dysfunction and decreases RV ROS levels at the same time; however, a causality between changes in ROS and preservation of RV morphology and/or function could not be proven.

In contrast to the pharmacological approaches, neither the genetic deletion of sirtuin 3 (Waypa et al., 2013) nor the up-regulation of thioredoxin 2 (Adesina et al., 2017) affected RV hypertrophy during pulmonary hypertension. Sirtuin-3 is a nicotinamide adenine dinucleotide–dependent deacetylase that activates forkhead box O3a (FOXO3)-dependent up-regulation of SOD-2 (Sundaresan et al., 2009). Thioredoxin 2 is a mitochondrial located protein involved in ROS defense of the organelle (Dunn et al., 2010).

β-Blockers may also considered as a therapeutic option in right heart failure. β-Adrencoeptor signaling is sensitized by ROS. At least in the left ventricle carvedilol, a β-blocker with antioxidative properties, was able to attenuate the hypertrophic response to anthracylines (Arozal et al., 2011). In rats with monocrotaline-induced pulmonary hypertension bucindolol treatment decreases RV necrosis, fibrosis, and infiltration of inflammatory cells and improves RV systolic function. In addition, bucindolol promotes a decrease in the cardiac sympathovagal balance by reducing sympathetic drive and increasing parasympathetic drive (Lima-Seolin et al., 2017). Changes in ROS were not measured. In a model of hypertension (two-kidney one-clip), β-blockers attenuated ROS and MMP2, a ROS-dependent regulated MMP, independent of its antioxidative property suggesting that direct stimulation of β-adrenoceptors increases ROS in ventricular tissue (Rizzi et al., 2014).

There are multiple reports that treatment regimes affecting the angiotensin-NOX-ROS axis attenuate hypertrophy and heart failure, but also few examples showing no effects (Table 1).

**Table 1:** Treatment of the angiotensin-NOX-ROS axis and effects on hypertrophy.

**Drug**	**Species**	**Tissue**	**Target**	**Read-out**	**References**
Isoflavone	Mice	LV	Ang-II-dependent	Hypertrophy	Chen et al., 2014
Taxofilin	Mice	LV	Ang-II-dependent	Hypertrophy	Guo et al., 2015
Spironolacton	Rats	LV	Renin-dependent	Hypertrophy	Habibi et al., 2011
Amlodipine/Atorvastatin	Rats	LV	Hypertension	Hypertrophy	Lu et al., 2009
Green Tea	Rats	LV	Ang-II-dependent	Hypertrophy	Papparella et al., 2008
AT1/ACE-I	Rats	LV	SHR	Hypertrophy	Tanaka et al., 2005
ACE inhibition	Rats	LV	Salt-induced BP	Cardiac function	Tsutsui et al., 2001
Atorvastatin	Rats	LV	Pressure overload	Hypertrophy	Li et al., 2013
Apocynin	Rats	LV	Pressure overload	Hypertrophy	Liu et al., 2010

## Conclusion

A coupling between ROS, cardiac hypertrophy and heart failure has been established for the LV. Concerning the RV only few data are available that directly analyzes right heart hypertrophy in the context of ROS signaling. As it stands there is consensus that RV tissue has a reduced oxidative defense capacity thereby favoring oxidative stress especially during the transition from RV hypertrophy to failure. Whether ROS targets in the RV include those proteins that are directly linked to cardiac growth is unclear and questionable. In contrast, oxidative modification of proteins leading to failure seems to be similar between both ventricles. Table 2 highlights the findings on ROS formation, defense, and targets in RV in comparison to LV.

**Table 2:** Differences between LV and RV in ROS handling leading to hypertrophy and failure.

**(A) ROS formation**			
	**NOX gp91**	**LV ↑**	**RV ↑**
	**NOX p47phox**	**LV ↑**	**RV ↑**
	**NOX2/4**		**RV ↑**
	**NOX-dependent ROS**	**LV ↑**
	**NOX-dependent Complex II**		**RV ↑**
	**XO**		**RV ↑**
	**NOS uncoupling**	**LV ↑**	**RV ↑**
	**PDK**		**RV ↑**
**(B) ROS defense**			
	**α-tocopherol nicotinate**		**RV ↓**
	**Non-oxidative glucose metabolism**		**RV ↑**
	**SOD-2**	**LV ↑**	**RV ↓**
	**PGC-1α**		**RV ↓**
	**Catalase**	**LV ↑**	
	**Glutathione peroxidase**	**LV ↑**
**(C) ROS-associated remodeling**			
	**AT-1 receptor**		**RV ↓**
	**ACE**		**RV ↑**
	**LOX-1**		**RV↑**

*↑, activated or induced during hypertrophy and/or transition to failure*.

*↓, deactivated or reduced during hypertrophy and/or transition to failure*.

## Author Contributions

K-DS and RaS wrote the manuscript, performed literature search, and work on the conception. HK and CH provided data to Figure 3 and read and improved the manuscript. RoS read and added conceptional ideas and data to the chapter defense system.

### Conflict of Interest Statement

The authors declare that the research was conducted in the absence of any commercial or financial relationships that could be construed as a potential conflict of interest.

## References

[B1] Abraham W. T. Raynolds M. V. Badesch D. B. Wynne K. M. Groves B. M. Roden R. L.. (2003). Angiotensin-converting enzyme DD genotype in patients with primary pulmonary hypertension: increased frequency and association with preserved haemodynamics. J. Renin. Angiot. Aldoster. Syst. 4, 27–30. 10.3317/jraas.2003.003 12692750

[B2] Adesina S. E. Wade B. E. Bijli K. M. Kang B. Y. Williams C. R. Ma J.. (2017). Hypoxia inhibits expression and function of mitochondrial thioredoxin 2 to promote pulmonary hypertension. Am. J. Physiol. Lung Cell. Mol. Physiol. 312, L599–L608. 10.1152/ajplung.00258.2016 28130258 PMC5451594

[B3] Alzoubi A. Toba M. Abe K. O'Neill K. D. Rocic P. Fagan K. A.. (2013). Dehydroepiandrosterone restores right ventricular structure and function in rats with severe pulmonary arterial hypertension. Am. J. Physiol. Heart Circ. Physiol. 304, H1708–H1718. 10.1152/ajpheart.00746.2012 23585128

[B4] Ameri P. Bertero E. Meliota G. Cheli M. Canepa M. Brunelli C.. (2016). Neurohormonal activation and pharmacological inhibition in pulmonary arterial hypertension and related right ventricular failure. Heart Fail. Rev. 21, 539–547. 10.1007/s10741-016-9566-3 27206576

[B5] Anghel N. Herman H. Balta C. Rosu M. Stan M. S. Nita D.. (2018). Acute cardiotoxicity induced by doxorubicin in right ventricle is associated with increase of oxidative stress and apoptosis in rats. Histol. Histopathol. 33, 365–378. 10.14670/HH-11-932 28920632

[B6] Arozal W. Sari F. R. Watanabe K. Arumugam S. Veeraveedu P. T. Ma M. (2011). Carvedilol-afforded protection against daunorubicin-induced cardiomyopathic rats *in vivo*: effects on cardiac fibrosis and hypertrophy. ISRN Pharmacol. 8:430549 10.5402/2011/430549 PMC3197008 22084713

[B7] Aziz A. Lee A. M. Ufere N. N. Damiano R. J. Townsend R. R. Moon M. R. (2015). Proteomic profiling of early chronic pulmonary hypertension: evidence for both adaptive and maladaptive pathology. J. Pulm. Respir. Med. 5:1000241. 10.4172/2161-105X.1000241 26246959 PMC4523278

[B8] Balestra G. M. Mik E. G. Eerbeek O. Specht P. A. van der Laarse W. J. Zuurbier C. J. (2015). Increased *in vivo* mitochondrial oxygenation with right ventricular failure induced by pulmonary arterial hypertension: mitochondrial inhibition as driver of cardiac failure? Respir. Res. 16:6. 10.1186/s12931-015-0178-6 25645252 PMC4320611

[B9] Borchi E. Bargelli V. Stillitano F. Giordano C. Sebastiani M. Nassi P. A.. (2010). Enhanced ROS production by NADPH oxidase is correlated to changes in antioxidant enzyme activity in human heart failure. Biochim. Biophys. Acta 1802, 331–338. 10.1016/j.bbadis.2009.10.014 19892017

[B10] Brittain E. L. Talati M. Fessel J. P. Zhu H. Penner N. Calcutt M. W.. (2016). Fatty acid metabolic defects and right ventricular lipotoxicity in human pulmonary arterial hypertension. Circulation 133, 1936–1944. 10.1161/CIRCULATIONAHA.115.019351 27006481 PMC4870107

[B11] Burger D. Montezano A. C. Nishgaki N. He Y. Carter A. Touyz R. M. (2011). Endothelial microparticle formation by angiotensin II is mediated via Ang II receptor type I/NADPH oxidase/Rho kinase pathways targeted to lipid rafts. Arterioscler. Thromb. Vasc. Biol. 31, 1898–1907. 10.1161/ATVBAHA.110.222703 21597004

[B12] Byrne J. A. Grieve D. J. Bendall J. K. Li J. M. Gove C. Lambeth J. D.. (2003). Contrasting roles of NADPH oxidase isoforms in pressure-overload versus angiotensin II–induced cardiac hypertrophy. Circ. Res. 93, 802–804. 10.1161/01.RES.0000099504.30207.F5 14551238

[B13] Cadenas S. (2018). Mitochondrial uncoupling, ROS generation and cardioprotection. Biochim. Biophys. Acta 1859, 940–950. 10.1016/j.bbabio.2018.05.019 29859845

[B14] Calamaras T. D. Lee C. Lan F. Ido Y. Siwik D. A. Colucci W. S. (2015). Lipid peroxidation product 4-hydroxy-trans-2-nonenal (HNE) causes protein synthesis in cardiac myocytes via activated mTORC1-P70S6K-RPS6 signaling. Free Radic. Biol. Med. 82, 137–146. 10.1016/j.freeradbiomed.2015.01.007 25617592 PMC4387097

[B15] Canton M. Menazza S. Sheeran F. L. Polverino de Laureto P. Di Lisa F. Pepe S.. (2011). Oxidation of myofibrillar proteins in human heart failure. J. Am. Coll. Cardiol. 57, 300–309. 10.1016/j.jacc.2010.06.058 21232667

[B16] Cau S. B. Barato R. C. Celes M. R. Muniz J. J. Rossi M. A. Tanus-Santos J. E. (2013). Doxycycline prevents acute pulmonary embolism-induced mortality and right ventricular deformation in rats. Cardiovasc. Drugs Ther. 27, 259–267. 10.1007/s10557-013-6458-9 23568586

[B17] Chai C. Z. Mo W. L. Zhuang X. F. Kou J. P. Yan Y. Q. Yu B. Y. (2016). Protective effects of Sheng-Mai-San on right ventricular dysfunction during chronic intermittent hypoxia in mice. Evid. Based Complement. Alternat. Med. 2016:4682786. 10.1155/2016/4682786 27073402 PMC4814671

[B18] Chang Y. M. Chang H. H. Lin H. J. Tsai C. C. Tsai C. T. Chang H. N.. (2017). Inhibition of cardiac hypertrophy effects in D-galactose-induced senescent hearts by alpinate oxyphyllae fructus treatment. Evid. Based Complement. Alternat. Med. 2017:2624384. 10.1155/2017/2624384 28479925 PMC5396449

[B19] Chemaly E. R. Hadri L. Zhang S. Kim M. Kohlbrenner E. Sheng J.. (2011). Long-term *in vivo* resistin overexpression induces myocardial dysfunction and remodeling in rats. J. Mol. Cell. Cardiol. 51, 144–155. 10.1016/j.yjmcc.2011.04.006 21549710 PMC3124590

[B20] Chen G. Pam S. Q. Chen C. Pan S. F. Zhang X. M. He Q. Y. (2014). Puerarin inhibits angiotensin II-induced cardiac hypertrophy via the redox-sensitive ERK1/2, p38 and NF-κB pathways. Acta Pharmacol. Sin. 35, 463–475. 10.1038/aps.2013.185 24608673 PMC4813721

[B21] Cheng Y. S. Dai D. Z. Dai Y. (2009). Isoproterenol disperses distribution of NADPH oxidase, MMP-9, and pPKCε in the heart, which are mitigated by endothelin receptor antagonist CPU0213. Acta Pharmacol. Sin. 30, 1099–1106. 10.1038/aps.2009.104 19597524 PMC4006683

[B22] Cingolani O. H. Pérez N. G. Ennis I. L. Alvarez M. C. Mosca S. M. Schinella G. R.. (2011). *In vivo* key role of reactive oxygen species and NHE-1 activation in determining excessive cardiac hypertrophy. Pflugers Arch. Eur. J. Physiol. 462, 733–743. 10.1007/s00424-011-1020-8 21870055

[B23] Ciulla M. M. Paliotti R. Carini M. Magrini F. Aldini G. (2011). Fibrosis, enzymatic and non-enzymatic cross-links in hypertensive heart disease. Cardiovasc. Hematol. Disord. Drug Targets 11, 61–73. 10.2174/187152911798347025 22044034

[B24] Cowley P. M. Wang G. Joshi S. Swigart P. M. Lovett D. H. Simpson P. C.. (2017). α1A-Subtype adrenergic agonist therapy for the failing right ventricle. Am. J. Physiol. Heart Circ. Physiol. 313, H1109–H1118. 10.1152/ajpheart.00153.2017 28822963 PMC5814653

[B25] Cruz J. A. Bauer E. M. Rodriguez A. I. Gangopadhyay A. Zeineh N. S. Wang Y.. (2012). Chronic hypoxia induces right heart failure in caveolin-1-/- mice. Am. J. Physiol. Heart Circ. Physiol. 302, H2518–H2527. 10.1152/ajpheart.01140.2011 22505641 PMC3378264

[B26] Danz E. D. Skramsted J. Henry N. Bennett J. A. Keller R. S. (2009). Resveratrol prevents doxorubicin cardiotoxicity through mitochondrial stabilization and the Sirt1 pathway. Free Radic. Biol. Med. 46, 1589–1597. 10.1016/j.freeradbiomed.2009.03.011 19303434

[B27] Date M. O. Morita T. Yamashita N. Nishida K. Yamaguchi O. Higuchi Y.. (2002). The antioxidant N-2-mercaptopropionyl glycine attenuates left ventricular hypertrophy in *in vivo* murine pressure-overload model. J. Am. Coll. Cardiol. 39, 907–912. 10.1016/S0735-1097(01)01826-5 11869860

[B28] Datta A. Kim G. A. Taylor J. M. Gugino S. F. Farrow K. N. Schumacker P. T.. (2015). Mouse lung development and NOX1 induction during hyperoxia are developmentally regulated and mitochondrial ROS dependent. Am. J. Physiol. Lung. Cell Mol. Physiol. 309, L369–L377. 10.1152/ajplung.00176.2014 26092998 PMC4587628

[B29] de Jong J. W. Schoemaker R. G. de Jonge R. Bernocchi P. Keijzer E. Harrison R.. (2000). Enhanced expression and activity of xanthine oxidoreductase in the failing heart. J. Mol. Cell. Cardiol. 32, 2083–2089. 10.1006/jmcc.2000.1240 11040111

[B30] DeMarco V. G. Habibi J. Whaley-Connell A. T. Schneider R. I. Heller R. L. Bosanquet J. P.. (2008). Oxidative stress contributes to pulmonary hypertension in the transgenic (mRen2)27 rat. Am. J. Physiol. Heart Circ. Physiol. 294, H2659–H2668. 10.1152/ajpheart.00953.2007 18424632

[B31] Dey S. DeMazumder D. Sidor A. Foster D. B. O'Rourke B. (2018). Mitochondrial ROS drive sudden cardiac death and chronic proteome remodeling in heart failure. Circ. Res. 123, 356–371. 10.1161/CIRCRESAHA.118.312708 29898892 PMC6057154

[B32] Di Lisa F. Giorgio M. Ferdinandy P. Schulz R. (2017). New aspects of p66Shc in ischaemia reperfusion injury and other cardiovascular diseases. Br. J. Pharmacol. 174, 1690–1703. 10.1111/bph.13478 26990284 PMC5446581

[B33] Dos Santos Lacerda D. Türck P. Gazzi de Lima-Seolin B. Colombo R. Duarte Ortiz V. Poletto Bonetto J. H.. (2017). Pterostilbene reduces oxidative stress, prevents hypertrophy and preserves systolic function of right ventricle in cor pulmonale model. Br. J. Pharmacol. 174, 3302–3314. 10.1111/bph.13948 28703274 PMC5595755

[B34] Duni A. Liakopoulos V. Rapsomanikis K. P. Dounousi E. (2017). Chronic kidney disease and disproportionally increased cardiovascular damage: does oxidative stress explain the burden? Oxidat. Med. Cell. Longevity. 2017:9036450. 10.1155/2017/9036450 29333213 PMC5733207

[B35] Dunn L. L. Buckle A. M. Cooke J. P. Ng M. K. (2010). The emerging role of the thioredoxin system in angiogenesis. Arterioscler. Thromb. Vasc. Biol. 30, 2089–2098. 10.1161/ATVBAHA.110.209643 20798378 PMC3142174

[B36] Ecarnot-Laubriet A. Rochette L. Vergely C. Sicard P. Teyssier J. R. (2003). The activation pattern of the antioxidant enzymes in the right ventricle of rat in response to pressure overload is of heart failure type. Heart Dis. 5, 308–312. 10.1097/01.hdx.0000089836.03515.a9 14503927

[B37] Egea J. Fabregat I. Frapart Y. M. Ghezzi P. Görlach A. Kietzmann T.. (2017). European contribution to the study of ROS: a summary of the findings and prospects for the future from the COST action BM1203 (EU-ROS). Redox Biol. 13, 94–162. 10.1016/j.redox.2017.05.007 28577489 PMC5458069

[B38] Frazziano G. Al Ghouleh I. Baust J. Shiva S. Champion H. C. Pagano P. J. (2014). Nox-derived ROS are acutely activated in pressure overload pulmonary hypertension: indications for a seminal role for mitochondrial Nox4. Am. J. Physiol. Heart Circ. Physiol. 306, H197–H205. 10.1152/ajpheart.00977.2012 24213612 PMC3920131

[B39] Freund-Michel V. Khoyrattee N. Savineau J. P. Muller B. Guibert C. (2014). Mitochondria: roles in pulmonary hypertension. Int. J. Biochem. Cell Biol. 55, 93–97. 10.1016/j.biocel.2014.08.012 25149415

[B40] Galhotra P. Prabhakar P. Meghwani H. Mohammed S. A. Banerjee S. K. Seth S. (2018). Beneficial effects of fenofibrate in pulmonary hypertension in rats. Mol. Cell. Biochem. 449, 185–194. 10.1007/s11010-018-3355-3 29761247

[B41] Gladden J. D. Zelickson B. R. Guichard J. L. Ahmed M. I. Yancey D. M. Ballinger S. (2013). Xanthine oxidase inhibition preserves left ventricular systolic but not diastolic function in cardiac volume overload. Am. J. Physiol. Heart Circ. Physiol. 305, H1440–H1450. 10.1152/ajpheart.00007.2013 24014679 PMC4073978

[B42] Gomez-Arroyo J. Mizuno S. Szczepanek K. Van Tassell B. Natarajan R. dos Remedios C. G.. (2013). Metabolic gene remodeling and mitochondrial dysfunction in failing right ventricular hypertrophy secondary to pulmonary arterial hypertension. Circ. Heart Fail. 6, 136–144. 10.1161/CIRCHEARTFAILURE.111.966127 23152488 PMC3790960

[B43] Grieve D. J. Byrne J. A. Siva A. Layland J. Johar S. Cave A. C.. (2006). Involvement of the nicotinamide adenosine dinucleotide phosphate oxidase isoform Nox2 in cardiac contractile dysfunction occurring in response to pressure overload. J. Am. Coll. Cardiol. 47, 817–826. 10.1016/j.jacc.2005.09.051 16487851

[B44] Guggilam A. Haque M. Kerut E. K. McIlwain E. Lucchesi P. Seghal I.. (2007). TNF-α blockade decreases oxidative stress in the paraventricular nucleus and attenuates sympathoexcitation in heart failure rats. Am. J. Physiol. Heart Circ. Physiol. 293, H599–H609. 10.1152/ajpheart.00286.2007 17416605

[B45] Guido M. C. Marques A. F. Tavares E. R. Tavares de Melo M. D. Salemi V. M. C. Maranhão R. C. (2017). The effects of diabetes induction on the rat heart: differences in oxidative stress, inflammatory cells, and fibrosis between subendocardial and interstitial myocardial areas. Oxidat. Med. Cell. Longevity 2017:5343972. 10.1155/2017/534397 28781721 PMC5525092

[B46] Guo H. Zhang X. Cui Y. Zhou H. Xu D. Shan T.. (2015). Taxifolin protects against cardiac hypertrophy and fibrosis during biomechanical stress of pressure overload. Toxicol. Appl. Pharmacol. 287, 168–177. 10.1016/j.taap.2015.06.002 26051872

[B47] Gupto A. A. Cordero-Reyes A. M. Youker K. A. Matsunami R. K. Engler D. A. Li S. (2016). Differential mitochondrial function in remodeled right and nonremodeled left ventricles in pulmonary hypertension. J. Card. Fail. 22, 73–81. 10.1016/j.cardfail.2015.09.001 26370778

[B48] Habibi J. DeMarco V. G. Ma L. Pulakat L. Rainey W. E. Whaley-Connell A. T.. (2011). Mineralocorticoid receptor blockade improves diastolic function independent of blood pressure reduction in a transgenic model of RAAS overexpression. Am. J. Physiol. Heart Circ. Physiol. 300, H1484–H1491. 10.1210/endo-meetings.2011.PART4.P2.P3-440 21239636 PMC3075026

[B49] Hansen T. Galougahi K. K. Celermajer D. Rasko N. Tang O. Bubb K. J.. (2016). Oxidative and nitrosative signalling in pulmonary arterial hypertension — implications for development of novel therapies. Pharmacol. Therap. 165, 50–62. 10.1016/j.pharmthera.2016.05.005 27216365

[B50] He J. Li X. Luo H. Li T. Zhao L. Qi Q.. (2017). Galectin-3 mediates the pulmonary arterial hypertension-induced right ventricular remodeling through interacting with NADPH oxidase 4. J. Am. Soc. Hypertens. 11, 275–289.e2. 10.1016/j.jash.2017.03.008 28431936

[B51] Hernandez-Resendiz S. Chinda K. Ong S. B. Cabrera-Fuentes H. Zazueta C. Hausenloy D. J. (2018). The role of redox dysregulation in the inflammatory response to acute myocardial ischaemia-reperfusion injury - adding fuel to the fire. Curr. Med. Chem. 25, 1275–1293. 10.2174/0929867324666170329100619 28356034

[B52] Heusch P. Aker S. Boengler K. Deindl E. van de Sand A. Klein K.. (2010). Increased inducible nitric oxide synthase and arginase II expression in heart failure: no net nitrite/nitrate production and protein S-nitrosylation. Am. J. Physiol. Heart Circ. Physiol. 299, H446–H453. 10.1152/ajpheart.01034.2009 20511413

[B53] Heusch P. Canton M. Aker S. van de Sand A. Konietzka I. Rassaf T.. (2010b). The contribution of reactive oxygen species and p38 mitogen-activated protein kinase to myofilament oxidation and progression of heart failure in rabbits. Br. J. Pharmacol. 160, 1408–1416. 10.1111/j.1476-5381.2010.00793.x 20590631 PMC2938812

[B54] Iacobazzi D. Suleiman M. S. Ghorbel M. George S. J. Caputo M. Tulloh R. M. (2016). Cellular and molecular basis of RV hypertrophy in congential heart disease. Heart 102, 12–17. 10.1136/heartjnl-2015-308348 26516182 PMC4717403

[B55] Ide T. Tsutsui H. Hayashidani S. Kang D. Suematsu N. Nakamura K.. (2001). Mitochondrial DNA damage and dysfunction associated with oxidative stress in failing hearts after myocardial Infarction. Circ. Res. 88, 529–535. 10.1161/01.RES.88.5.529 11249877

[B56] Ishikawa K. Kimura S. Kobayashi A. Sato T. Matsumoto H. Ujiie Y.. (2005). Increased reactive oxygen species and anti-oxidative response in mitochondrial cardiomyopathy. Circ. J. 69, 617–620. 10.1253/circj.69.617 15849452

[B57] Karamanlidis G. Bautista-Hernandez V. Fynn-Thompson F. Del Nido P. Tian R. (2011). Impaired mitochondrial biogenesis precedes heart failure in right ventricular hypertrophy in congenital heart disease. Circ. Heart Fail. 4, 707–713. 10.1161/CIRCHEARTFAILURE.111.961474 21840936 PMC3218261

[B58] Koga K. Kenessey A. Ojamaa K. (2013). Macrophage migration inhibitory factor antagonizes pressure overload-induced cardiac hypertrophy. Am. J. Physiol. Heart Circ. Physiol. 304, H282–H293. 10.1152/ajpheart.00595.2012 23144312

[B59] Li J. M. Gall N. P. Grieve D. J. Chen M. Shah A. M. (2002). Activation of NADPH oxidase during progression of cardiac hypertrophy to failure. Hypertension 40, 477–484. 10.1161/01.HYP.0000032031.30374.32 12364350

[B60] Li M. Sala V. De Santis M. C. Cimino J. Cappello P. Pianca N.. (2018). Phosphoinositide 3-kinase gamma inhibition protects from anthracycline cardiotoxicity and reduces tumor growth. Circulation 138, 696–711. 10.1161/CIRCULATIONAHA.117.030352 29348263

[B61] Li R. Fang W. Cao S. Li Y. Wang J. Xi S. (2013). Differential expression of NAD(P)H oxidase isoforms and the effect of atorvastatin on cardiac remodeling in two-kidney two-clip hypertensive rats. Pharmazie 68, 261–269. 10.1691/ph.2013.2782 23700792

[B62] Lima-Seolin B. G. Colombo R. Bonetto J. H. P. Teixeira R. B. Donatti L. M. Casali K. R.. (2017). Bucindolol improves right ventricle function in rats with pulmonary arterial hypertension through the reversal of autonomic imbalance. Eur. J. Pharmacol. 798, 57–65. 10.1016/j.ejphar.2016.12.028 28011346

[B63] Liu B. Luo X. J. Yang Z. B. Zhang J. J. Li T. B. Zhang X. J.. (2014). Inhibition of NOX/VPO1 pathway and inflammatory reaction by trimethoxystilbene in prevention of cardiovascular remodeling in hypoxia-induced pulmonary hypertensive rats. J. Cardiovasc. Pharmacol. 63, 567–576. 10.1097/FJC.0000000000000082 24492474

[B64] Liu J. Zhou J. An W. Lin Y. Yang Y. Zang W. (2010). Apocynin attenuates pressure overload-induced cardiac hypertrophy in rats by reducing levels of reactive oxygen species. Can. J. Physiol.Pharmacol. 88, 745–752. 10.1139/Y10-063 20651822

[B65] Liu J. Q. Zelko I. N. Erbynn E. M. Sham J. S. Folz R. J. (2006). Hypoxic pulmonary hypertension: role of superoxide and NADPH oxidase (gp91phox). Am. J. Physiol. Lung Cell. Mol. Physiol. 290, L2–L10. 10.1152/ajplung.00135.2005 16085672

[B66] Liu L. Marcocci L. Wong C. M. Park A. M. Suzuki Y. J. (2008). Serotonin-mediated protein carbonylation in the right heart. Free Radic. Biol. Med. 45, 847–854. 10.1016/j.freeradbiomed.2008.06.008 18616998 PMC2574542

[B67] Lu J. C. Cui W. Zhang H. L. Liu F. Han M. Liu D. M.. (2009). Additive beneficial effects of amilodipine and atorvastatin in reversing advanced cardiac hypertophy in elderly spontaneously hypertensive rats. Clin. Exptl. Pharmacol. Physiol. 36, 1110–1119. 10.1111/j.1440-1681.2009.05198.x 19413592

[B68] Lu X. Dang C. Q. Guo X. Molloi S. Wassall C. D. Kemple M. D.. (2011). Elevated oxidative stress and endothelial dysfunction in right coronary artery of right ventricular hypertrophy. J. Appl. Physiol. 110, 1674–1681. 10.1152/japplphysiol.00744.2009 21415175 PMC3119132

[B69] Lu Z. Xu X. Hu X. Fassett J. Zhu G. Tao Y. (2010). PGC-1a regulates expression of myocardial mitochondrial antioxidants and myocardial oxidative stress after chronic systolic overload. Antiox. Redox. Signal. 13, 1011–1022. 10.1089/ars.2009.2940 PMC2959178 20406135

[B70] Lüneburg N. Siques P. Brito J. Arriaza K. Pena E. Klose H.. (2016). Long-term chronic intermittent hypobaric hypoxia in rats causes an imbalance in the asymmetric dimethylarginine/nitric oxide pathway and ROS activity: a possible synergistic for altitude pulmonary hypertension? Pulm. Med. 2016:6578578. 10.1155/2016/6578578 27313889 PMC4904121

[B71] Ma L. Ambalavanan N. Liu H. Sun Y. Jhala N. Bradley W. E. (2016). TLR4 regulates pulmonary vascular homeostasis and remodeling via redox signaling. Front. Biosci. 21, 397–409. 10.2741/4396 26709781 PMC4706231

[B72] Maarman G. J. Schulz R. Sliwa K. Schermuly R. T. Lecour S. (2017). Novel putative pharmacological therapies to protect the right ventricle in pulmonary hypertension: a review of current literature. Br. J. Pharmacol. 174, 497–511. 10.1111/bph.13721 28099680 PMC5345550

[B73] Mandraffino G. Aragona C. O. Cairo V. Scuruchi M. Lo Gullo A. D'ascola A.. (2017). Circulating progenitor cells in hypertensive subjects: effectiveness of a treatment with olmesartan in improving cell number and miR profile in addition to expected pharmacological effects. PLoS ONE 12:e0173030. 10.1371/journal.pone.0173030 28301500 PMC5354372

[B74] Manni M. E. Rigacci S. Borchi E. Bargelli V. Miceli C. Giordano C.. (2016). Monoamine oxidase is overactivated in left and right ventricles from ischemic hearts: an intriguing therapeutic target. Oxidat. Med. Cell. Longevity 2016:4375418. 10.1155/2016/4375418 28044091 PMC5156804

[B75] Matsuda S. Umemoto S. Yoshimura K. Itoh S. Murata T. Fukai T. (2015). Angiotensin II activates MCP-1 and induces cardiac hypertrophy and dysfunction via Toll-like receptor 4. Atheroscler. Thromb. 26, 833–844. 10.5551/jat.27292 PMC5509415 25752363

[B76] Moris D. Spartalis M. Tzatzaki E. Spartalis E. Karachaliou G. S. Triantafyllis A. S.. (2017). The role of reactive oxygen species in myocardial redox signaling and regulation. Ann. Transl. Med. 5:324. 10.21037/atm.2017.06.17 28861421 PMC5566737

[B77] Mouli S. Nanayakkara G. AlAlasmari A. Eldoumani H. Fu X. Berlin A.. (2015). The role of frataxin in doxorubicin-mediated cardiac hypertrophy. Am. J. Physiol. Heart Circ. Physiol. 309, H844–H859. 10.1152/ajpheart.00182.2015 26209053

[B78] Nagendran J. Gurtu V. Fu D. Z. Dyck J. R. Haromy A. Ross D. B.. (2008). A dynamic and chamber-specific mitochondrial remodeling in right ventricular hypertrophy can be therapeutically targeted. J. Thorac. Cardiovasc. Surg. 136, 168–178. 10.1016/j.jtcvs.2008.01.040 18603070

[B79] Nediani C. Borchi E. Giordano C. Baruzzo S. Ponziani V. Sebastiani M.. (2007). NADPH oxidase-dependent redox signaling in human heart failure: relationship between the left and right ventricle. J. Mol. Cell. Cardiol. 42, 826–834. 10.1016/j.yjmcc.2007.01.009 17346742

[B80] Nisbet R. E. Graves A. S. Kleinhenz D. J. Rupnow H. L. Reed A. L. Fan T. H.. (2009). The role of NADPH oxidase in chronic intermittent hypoxia-induced pulmonary hypertension in mice. Am. J. Respir. Cell Mol. Biol. 40, 601–609. 10.1165/2008-0145OC 18952568 PMC2677439

[B81] Ogura S. Shimosawa T. Mu S. Sonobe T. Kawakami-Mori F. Wang H.. (2013). Oxidative stress augments pulmonary hypertension in chronically hypoxic mice overexpressing the oxidized LDL receptor. Am. J. Physiol. Heart Circ. Physiol. 305, H155–H162. 10.1152/ajpheart.00169.2012 23686713

[B82] Papparella I. Ceolotto G. Montemurro D. Antonello M. Garbisa S. Rossi G. P. (2008). Green tea attenuates angiotensin II-induced cardiac hypertrophy in rats by modulating reactive oxygen species production and the Src/Epidermal growth factor receptor/Act signalling pathway. J. Nutr. 138, 1596–1601. 10.1093/jn/138.9.1596 18716156

[B83] Paulin R. Michelakis E. D. (2014). The metabolic theory of pulmonary arterial hypertension. Circ Res. 115, 148–164. 10.1161/CIRCRESAHA.115.301130 24951764

[B84] Phillips D. Aponte A. M. Covian R. Neufeld E. Yu Z. X. Balaban R. S. (2011). Homogenous protein programming in the mammalian left and right ventricle free walls. Physiol. Genom. 43, 1198–1206. 10.1152/physiolgenomics.00121.2011 21878611 PMC3217320

[B85] Pichardo J. Palace V. Farahmand F. Singal P. K. (1999). Myocardial oxidative stress changes during compensated right heart failure in rats. Mol. Cell. Biochem. 196, 51–57. 10.1023/a:1006914111957 10448902

[B86] Pönicke K. Schlüter K. D. Heinroth-Hoffmann I. Seyfarth T. Goldberg M. Piper H. M. (2001). Naunyn Schmiedebergs. *Arch*. Pharmacol. 364, 444–453. 10.1007/s002100100469 11692228

[B87] Puukila S. Fernandes R. O. Türck P. Carraro C. C. Bonetto J. H. P. de Lima-Seolin B. G.. (2017). Secoisolariciresinol diglucoside attenuates cardiac hypertrophy and oxidative stress in monocrotaline-induced right heart dysfunction. Mol. Cell. Biochem. 432, 33–39. 10.1007/s11010-017-2995-z 28321539

[B88] Qiao W. Zhang W. Gai Y. Zhao L. Fan J. (2014). The histone acetyltransferase MOF overexpression blunts cardiac hypertrophy by targeting ROS in mice. Biochem. Biophys. Res. Commun. 448, 379–384. 10.1016/j.bbrc.2014.04.112 24802406

[B89] Qin F. Siwik D. A. Pimentel D. R. Morgan R. J. Biolo A. Tu V. H.. (2017). Cytosolic H_2_O_2_ mediates hypertrophy, apoptosis, and decreased SERCA activity in mice with chronic hemodynamic overload. Am. J. Physiol. Heart Circ. Physiol. 306, H1453–H1463. 10.1152/ajpheart.00084.2014 24633550 PMC4024717

[B90] Qipshidze N. Tyagi N. Metreveli N. Lominadze D. Tyagi S. C. (2012). Autophagy mechanism of right ventricular remodeling in murine model of pulmonary artery constriction. Am. J. Physiol. Heart Circ. Physiol. 302, H688–H696. 10.1152/ajpheart.00777.2011 22101525 PMC3353777

[B91] Rassaf T. Poll L. W. Brouzos P. Lauer T. Totzeck M. Kleinbongard P.. (2006). Positive effects of nitric oxide on left ventricular function in humans. Eur. Heart J. 27, 1699–1705. 10.1093/eurheartj/ehl096 16782717

[B92] Rawat D. K. Alzoubi A. Gupte R. Chettimada S. Watanabe M. Kahn A. G.. (2014). Increased reactive oxygen species, metabolic maladaptation, and autophagy contribute to pulmonary arterial hypertension-induced ventricular hypertrophy and diastolic heart failure. Hypertension 64, 1266–1274. 10.1161/HYPERTENSIONAHA.114.03261 25267798

[B93] Reddy S. Bernstein D. (2015). Molecular mechanisms of right ventricular failure. Circulation 132, 1734–1742. 10.1161/CIRCULATIONAHA.114.012975 26527692 PMC4635965

[B94] Redout E. M. van der Toorn A. Zuidwijk M. J. van de Kolk C. W. A. van Echteld C. J. A. Musters R. J. P.. (2010). Antioxidant treatment attenuates pulmonary arterial hypertension-induced heart failure. Am. J. Physiol. Heart Circ. Physiol. 298, H1038–H1047. 10.1152/ajpheart.00097.2009 20061549

[B95] Redout E. M. Wagner M. J. Zuidwijk M. J. Boer C. Musters R. J. P. van Hardeveld C.. (2007). Right-ventricular failure is associated with increased mitochondrial complex II activity and production of reactive oxygen species. Cardiovasc. Res. 75, 770–781. 10.1016/j.cardiores.2007.05.012 17582388

[B96] Redza-Dutordoir M. Averill-Bates D. A. (2016). Activation of apoptosis signalling pathways by reactive oxygen species. Biochim. Biophys. Acta 1863, 2977–2992. 10.1016/j.bbamcr.2016.09.012 27646922

[B97] Riba A. Deres L. Sumegi B. Toth K. Szabados E. Halmosi R. (2017). Cardioprotective effect of resveratrol in a postinfarction heart failure model. Oxidative Med. Cell. Longevity 2017:6819281. 10.1155/2017/6819281 29109832 PMC5646324

[B98] Rizzi E. Guimaraes D. A. Ceron C. S. Prado C. M. Pinheiro L. C. Martins-Oliveira A.. (2014). β1-Adrenergic blockers exert antioxidant effects, reduce matrix metalloproteinase activity, and improve renovascular hypertension-induced cardiac hypertrophy. Free Radical. Biol. Med. 73, 308–317. 10.1016/j.freeradbiomed.2014.05.024 24933619

[B99] Schäfer M. Schäfer C. Piper H. M. Schlüter K. D. (2002). Hypertrophic responsiveness of cardiomyocytes to α- and β-adrenoceptor stimulation requires sodium-proton-exchanger-1 (NHE-1) activation but not cellular alkalization. Eur. J. Heart Fail. 4, 249–254. 10.1016/S1388-9842(02)00016-8 12034148

[B100] Schlüter K. D. (ed.). (2016). Ways to study the biology of cardiomyocytes, in Cardiomyocytes – Active Players in Cardiac Disease (Cham: Springer International Publishing), 3–23. 10.1007/978-3-319-31251-4_1

[B101] Schreckenberg R. Rebelo M. Deten A. Weber M. Rohrbach S. Pipicz M.. (2015). Specific mechanisms underlying right heart failure: the missing upregulation of superoxide dismutase-2 and its decisive role in antioxidative defense. Antioxdi. Redox Signal. 23, 1220–1232. 10.1089/ars.2014.6139 25978844 PMC4657518

[B102] Shimizu S. Ishibashi M. Kumagai S. Wajima T. Hiroi T. Kurihara T.. (2013). Decreased cardiac mitochondrial tetrahydrobiopterin in a rat model of pressure overload. Int. J. Mol. Med. 31, 589–596. 10.3892/ijmm.2013.1236 23313998

[B103] Simm A. Schlüter K. D. Diez C. Piper H. M. Hoppe J. (1998). Activation of p70S6 kinase by β-adrenoceptor agonists on adult cardiomyocytes. J. Mol. Cell. Cardiol. 30, 2059–2067. 10.1006/jmcc.1998.0768 9799659

[B104] Siqueira R. Colombo R. Conzatti A. de Castro A. L. Carraro C. C. Tavares A. M. V.. (2018). Effects of ovariectomy on antioxidant defence systems in the right ventricle of female rats with pulmonary arterial hypertension induced by monocrotaline. Can. J. Physiol. Pharmacol. 96, 295–303. 10.1139/cjpp-2016-0445 28854338

[B105] Sirker A. Murdoch C. E. Protti A. Sawyer G. J. Santos C. X. C. Martin D.. (2016). Cell-specific effects of Nox2 on the acute and chronic response to myocardial infarction. J. Mol. Cell. Cardiol. 98, 11–17. 10.1016/j.yjmcc.2016.07.003 27397876 PMC5029266

[B106] Sundaresan N. R. Gupta M. Kim G. Rajamohan S. B. Isbatan A. Gupta M. P. (2009). Sirt3 blocks the cardiac hypertrophic response by augmenting Foxo3a-dependent antioxidant defense mechanisms in mice. J. Clin. Invest. 119, 2758–2771. 10.1172/JCI39162 19652361 PMC2735933

[B107] Sutendra G. Michelakis E. D. (2014). The metabolic basis of pulmonary arterial hypertension. Cell Metab. 19, 558–573. 10.1016/j.cmet.2014.01.004 24508506

[B108] Sverdlov A. L. Elezaby A. Qin F. Behring J. B. Luptak I. Calamaras T. D.. (2016). Mitochondrial reactive oxygen species mediate cardiac structural, functional, and mitochondrial consequences of diet-induced metabolic heart disease. J. Am. Heart. Assoc. 5:e002555. 10.1161/JAHA.115.002555 26755553 PMC4859372

[B109] Takimoto E. Champion H. C. Li M. Ren S. Rodriguez E. R. Tavazzi B.. (2005). Oxidant stress from nitric oxide synthase−3 uncoupling stimulates cardiac pathologic remodeling from chronic pressure load. J. Clin. Invest. 115, 1221–1231. 10.1172/JCI21968 15841206 PMC1077169

[B110] Talukder M. A. Johnson W. M. Varadharaj S. Lian J. Kearns P. N. El-Mahdy M. A.. (2011). Chronic cigarette smoking causes hypertension, increased oxidative stress, impaired NO bioavailability, endothelial dysfunction, and cardiac remodeling in mice. Am. J. Physiol. Heart Circ. Physiol. 300, H388–H396. 10.1152/ajpheart.00868.2010 21057039 PMC3023256

[B111] Tanaka M. Umemoto S. Kawahara S. Kubo M. Itoh S. Umeji K.. (2005). Angiotensin II type 1 receptor antagonist and angiotensin-converting enzyme inhibitor altered the activation of Cu/Zn-containing superoxide dismutase in the heart of stroke-prone spontaneously hypertensive rats. Hypertens. Res. 28, 67–77. 10.1291/hypres.28.67 15969257

[B112] Tsutsui H. Ide T. Hayashidami S. Kinugawa S. Suematsu N. Utsumi H.. (2001). Effects of ACE inhibition on left ventricular failure and oxidative stress in Dahl salt-sensitive rats. J. Cardiovasc. Pharmacol. 37, 725–733. 10.1097/00005344-200106000-00010 11392469

[B113] Türck P. Lacerda D. S. Carraro C. C. de Lima-Seolin B. G. Teixeira R. B. Poletto Bonetto J. H.. (2018). Trapidil improves hemodynamic, echocardiographic and redox state parameters of right ventricle in monocrotaline-induced pulmonary arterial hypertension model. Biomed. Pharmacother. 103, 182–190. 10.1016/j.biopha.2018.04.001 29653363

[B114] Umemoto S. Tanaka M. Kawahara S. Kubo M. Umeji K. Hashimoto R.. (2004). Calcium antagonist reduces oxidative stress by upregulating Cu/Zn superoxide dismutase in stroke-prone spontaneously hypertensive rats. Hypertens. Res. 27, 877–885. 10.1291/hypres.27.877 15824470

[B115] Wang L. Lopaschuk G. D. Clanachan A. S. (2008). H_2_O_2_-induced left ventricular dysfunction in isolated working rat hearts is independent of calcium accumulation. J. Mol. Cell. Cardiol. 45, 787–795. 10.1016/j.yjmcc.2008.08.010 18817782

[B116] Wang X. Liu J. Drummond C. A. Shapiro J. I. (2017a). Sodium potassium adenosine triphosphatase (Na/K-ATPase) as a therapeutic target for uremic cardiomyopathy. Exp. Opin. Ther. Targets 21, 531–541. 10.1080/14728222.2017.1311864 28338377 PMC5590225

[B117] Wang X. Shults N. V. Suzuki Y. J. (2017). Oxidative profiling of the failing right heart in rats with pulmonary hypertension. PLoS ONE 12:e0176887. 10.1371/journal.pone.0176887 28472095 PMC5417519

[B118] Waypa G. B. Osborne S. W. Marks J. D. Berkelhamer S. K. Kondapalli J. Schumacker P. T. (2013). Sirtuin 3 deficiency does not augment hypoxia-induced pulmonary hypertension. Am. J. Respir. Cell Mol. Biol. 49, 885–891. 10.1165/rcmb.2013-0191OC 24047466 PMC3931121

[B119] Wenzel S. Abdallah Y. Helmig S. Schäfer C. Piper H. M. Schlüter K.-D. (2006). Contribution of PI 3-kinase isoforms to angiotzensin II- and α-adrenoceptor-mediated signaling pathways in cardiomyocytes. Cardiovasc. Res. 71, 352–362. 10.1016/j.cardiores.2006.02.004 16750184

[B120] Wenzel S. Rohde C. Wingerning S. Roth J. Kojda G. Schlüter K. D. (2007). Lack of endothelial nitric oxide synthase-derived nitric oxide formation favors hypertrophy in adult ventricular cardiomyocytes. Hypertension 49, 193–200. 10.1161/01.HYP.0000250468.02084.ce 17075027

[B121] Wenzel S. Taimor G. Piper H. M. Schlüter K. D. (2001). Redox-sensitive intermediates mediate angiotensin II-induced p38 MAP kinase activation, AP-1 binding activity, and TGF-β expression in adult ventricular cardiomyocytes. FASEB J. 15, 2291–2293. 10.1096/fj.00-0827fje 11511516

[B122] Wong C. M. Bansal G. Pavlickova L. Marocci L. Suzuki Y. J. (2013). Reactive oxygen species and antioxidants in pulmonary hypertension. Antioxid. Redox Signal. 18, 1789–1796. 10.1089/ars.2012.4568 22657091 PMC3619148

[B123] Wong Y. Y. Ruiter G. Lubberink M. Raijmakers P. G. Knaapen P. Marcus J. T.. (2011). Right ventricular failure in idiopathic pulmonary arterial hypertension is associated with inefficient myocardial oxygen utilization. Circ. Heart Fail. 4, 700–706. 10.1161/CIRCHEARTFAILURE.111.962381 21900188

[B124] Xu Q. Dalic A. Fang L. Kiriazis H. Ritchie R. H. Sim K. (2011). Myocardial oxidative stress contributes to transgenic β_2_-adrenoceptor activation induced cardiomyopathy and heart failure. Br. J. Pharmacol. 162, 1012–1028. 10.1111/j.1476-5381.2010.01043.x 20955367 PMC3051376

[B125] Ye J. X. Wang S. S. Ge M. Wang D. J. (2016). Suppression of endothelial PGC-1α is associated with hypoxia-induced endothelial dysfunction and provides a new therapeutic target in pulmonary arterial hypertension. Am. J. Physiol. Lung. Cell Mol. Physiol. 310, L1233–L1242. 10.1152/ajplung.00356.2015 27084848

[B126] Zhu T. T. Zhang W. F. Luo P. Qian Z. X. Li F. Zhang Z.. (2017). LOX-1 promotes right ventricular hypertrophy in hypoxia-exposed rats. Life Sci. 174, 35–42. 10.1016/j.lfs.2017.02.016 28259654

[B127] Zisman L. S. Asano K. Dutcher D. L. Ferdensi A. Robertson A. D. Jenkin M.. (1998). Differential regulation of cardiac angiotensin converting enzyme binding sites and AT_1_ receptor density in the failing human heart. Circulation 98, 1735–1741. 10.1161/01.CIR.98.17.1735 9788827

